# A Novel Protein Sourced from Chinese Medicine Residue for Golden Pompano Feed: Endothelium Corneum Gigeriae Galli Residue (ECGGR)

**DOI:** 10.1155/2024/1845188

**Published:** 2024-05-13

**Authors:** Ziqiao Wang, Rong Yao, Xuanshu He, Xin Cui, Zhihong Liao, Yantao Liu, Hanlin Wei, Zhenxiao Zhuang, Mengdie Chen, Jin Niu

**Affiliations:** State Key Laboratory of Biocontrol, Guangdong Provincial Key Laboratory for Aquatic Economic Animals and Southern Marine Science and Engineering Guangdong Laboratory (Zhuhai), School of Life Sciences, Sun Yat-Sen University, Guangzhou, China

## Abstract

Fishmeal is an important protein source in aquafeed. However, due to the limited natural resources, fishmeal is in short supply, resulting in a price surge for fishmeal. Here, we reported a kind of Chinese medicine residue, endothelium corneum gigeriae galli residue (ECGGR), as a fishmeal substitute in the diets of *Trachinotus ovatus*. Six isonitrogenous and isolipidic diets were formulated, substituting fishmeal at 0%, 6.25%, 12.5%, 18.75%, 25%, and 31.25%. There was no significant difference in the growth performance when the fishmeal substitution level was no more than 25%. The smallest FCR was obtained at the 18.75% substitution level. Furthermore, substituting ECGGR for fishmeal had no effect on whole-body and muscle proximate compositions, except when the replacement level exceeded 25%, which led to a decrease in whole-body moisture and an increase in whole-body crude protein. The contents of Gly, Cys, Ile, Tyr, Pro, and EAAs/TAAs were altered as the substitution level varied. However, dietary replacement of fishmeal with ECGGR did not degrade muscle protein quality, according to a nutritional evaluation of muscle essential amino acid composition. In terms of hepatic antioxidant capacity, neither the overall antioxidant status nor the expression of genes in the Nrf2-ARE pathway was altered by dietary ECGGR. Moreover, the expressions of *p65*, *TNF-α*, and *IL-8* in the intestine were upregulated at the 31.25% substitution level. Also, more goblet cells were observed in the intestine at substitution levels of 25% and 31.25%. In conclusion, ECGGR can substitute for fishmeal at the optimal level of 18.75% without adversely affecting the growth performance, protein quality, or hepatic and intestinal health of golden pompano.

## 1. Introduction

Booming in recent decades, the production of aquaculture has risen from 14.9 million metric tons in 1986 to 87.5 million metric tons in 2020, representing 49% of the total production of world fisheries and aquaculture [1, 2]. Considered the most nutritious and digestible ingredient for farmed fish, fishmeal was utilized as the primary protein source in many carnivorous fish feeds, leading to a huge demand for fishmeal. In 2020, approximately 9% (over 16 million metric tons) of global fish production was used to generate fishmeal and fish oil for aquafeed [1]. However, limited by the slow-growing fisheries industry, the supply of fishmeal cannot satisfy the surging demand of the aquaculture industry, which has, therefore, pushed up the price of fishmeal. Thus, it is urgent to explore low-cost and sustainable alternative protein sources. Some protein sources have been demonstrated to be capable of partially substituting for fishmeal, such as poultry byproduct meal (PBM), feather meal, soybean meal, rapeseed meal, cottonseed meal, *Methylococcus capsulatus* protein, *Clostridium autoethanogenum* protein, and so on [3–11].

Endothelium corneum gigeriae galli (ECGG) is the dried inner membrane of the gizzard of *Gallus gallus domesticus* Brisson. It is described as a well-known traditional Chinese medicine used to treat lithiasis in the Chinese pharmacopeia [12]. Some chemical components extracted from ECGG, such as polysaccharides and soy isoflavones, were demonstrated to possess antioxidant, antiurolithic, and cardioprotective properties [13–16]. Xiangbing et al. [17] reported that polysaccharides from ECGG could improve the digestive capacity, antioxidant capacity, and serum biochemical indices of juvenile *L. calcarifer*. Endothelium corneum gigeriae galli residue (ECGGR) is leftover after these effective components are extracted by pharmaceutical companies. ECGGR is not only a Chinese medicine residue but also a chicken byproduct, which means it contains high crude protein content and has the potential for substituting for fishmeal. Currently, there is a scarcity of research on viscera as a fishmeal substitute in aquafeed. It was reported that chicken intestinal hydrolysates could replace 50% of fishmeal and did not significantly affect the growth performance and intestinal immunity of common carp, *Cyprinus carpio* [18]. Enzyme-digested hydrolyzed porcine mucosa was used to substitute for 9.1% fishmeal without negative effects on growth as well as the digestive and absorptive functions of the intestines in the hybrid grouper *Epinephelus fuscoguttatus* ♀ × *E. lanceolatus* ♂ [19].

*Trachinotus ovatus* is an important commercial species of marine fish farmed throughout tropical and temperate waters worldwide [20]. On account of its fast growth and great taste, it is extensively farmed on the southeast coast of China, with an annual production of 2.45 × 10^6^ t in 2022 [21]. As a carnivorous fish, the formula of *T. ovatus* usually consists of more than 30% fishmeal, which is incompatible with the sustained growth of aquaculture [22]. The objective of this project was to evaluate the potential of ECGGR as a fishmeal alternative in juvenile *T. ovatus* diets.

## 2. Materials and Methods

### 2.1. Experimental Diet Preparation

ECGGR was provided by Guangdong Yifang Pharmaceutical Co., Ltd. The ECGGR contained 83.93% crude protein and 1.59% crude lipid, and its amino acid profile was also displayed in Table 1.

To formulate the experimental diets, fishmeal was substituted by ECGGR at gradient levels of 0% (TD1), 6.25% (TD2), 12.5% (TD3), 18.75% (TD4), 25% (TD5), and 31.25% (TD6). Soybean meal, soy protein isolate, and soy oil were added in varying amounts to ensure that the test diets were isonitrogenous (42%) and isolipidic (15%) (Table 2). All dry ingredients were triturated and sifted through a 60-mesh screen. After being weighed, these ingredients were thoroughly mixed in the VALVA-30-1S mixer (VALVA Machinery Equipment Co., Ltd., Guangzhou, China). Distilled water (about 35%, v/w) was added to the machine and mixed with the ingredient powders. The wet mixture was transferred to the VALVA60-III Twin Screw Extruder and processed as 2.5-mm pellets. After the extrusion, the extruded product was collected and naturally air-dried at 16°C until the moisture decreased to approximately 100 g/kg. The experimental feeds were packaged in plastic bags and refrigerated at −20°C until used.

### 2.2. Fish and Experimental Setup

Juvenile *T. ovatus* were purchased from a local breeding farm (Lingshui, Hainan, China). Prior to the experiment, the fish were acclimated to the experimental conditions (water temperature 30°C, pH 8.0–8.4, DO ≥ 7.0 mg/L) and fed with the control diet for 2 weeks. Then, golden pompano juveniles (initial body weight, 13.22 ± 0.26 g) were randomly distributed into 18 net cages (1.1 m × 1.1 m × 1.6 m, 30 fish per cage, triplicate cages per treatment). Fish were fed twice daily, at 8:30 and 16:30, for 53 consecutive days. During the breeding process, the feed intake and mortality of fish were documented.

### 2.3. Sample Collection

After a 53-day breeding experiment, all fish were fasted for 24 hr and then anesthetized, weighed, and counted. A randomized sample of four fish from each cage was taken and preserved at −30°C for chemical analysis. Four fish from each cage were individually weighed, quantified for body length, and dissected subsequently. Their viscera and liver were weighed, and the muscles were harvested for analysis of proximate composition and amino acids. Meanwhile, the livers and intestines were instantly removed, frozen in liquid nitrogen, and preserved at −80°C for analysis of antioxidant status and inflammation. Besides, the mid-intestinal segments were fixed in 4% paraformaldehyde for the assessment of intestinal morphology.

### 2.4. Chemical Analysis of Feed, Whole Body, and Muscle

The proximate composition was analyzed according to the standard methodology of the Association of Official Analytical Chemists [23]. To quantify the moisture content, the samples were dehydrated at 105°C to a constant weight. The Dumas method was employed to evaluate crude protein (*N* × 6.25) (DUMATHREM® DT N Pro, Gerhart, Germany). Crude lipid was measured based on the Soxhlet method (Soxtec System HT6, Tecator, Sweden). The muscle samples were freeze-dried by a freeze drier (LC-10N-50A, LICHEN, Shanghai, China) before the quantification of amino acid contents (S-433 Amino Acid Analyzer, Sykam, Germany).

### 2.5. Hepatic Antioxidant Status Analysis

Livers were homogenized with ice-cold PBS (1 : 9, w/v) and centrifuged at 2,000 rpm for 10 min at 4°C to obtain the supernatant. Total superoxide dismutase (T-SOD), catalase (CAT), glutathione peroxidase (GSH-PX), total antioxidant capacity (T-AOC), and malondialdehyde (MDA) were quantified with assay kits (Nanjing Jian Cheng Bioengineering Institute, China) following the protocols.

### 2.6. Reverse Transcription-Quantitative PCR

The livers and intestines from the same treatment were pooled, respectively. Total RNA was isolated using a commercial kit (R0027, Beyotime Biotechnology, China). Subsequently, cDNA was synthesized with the *Evo M-MLV* RT mix kit (AG, China). The primer sequences are listed in Table 3.

Real-time PCR was performed on a Light Cycler 480 (Roche Applied Science, Basel, Switzerland) using a SYBR Green *Pro Taq* HS qPCR kit (AG, China) following the manufacturer's protocol. Gene expression quantities were normalized to *beta-actin*, and the 2^−*ΔΔ*Ct^ method was applied to the calculation of relative expression levels.

### 2.7. Intestine Histological Observation

The intestinal samples were dehydrated in gradient ethanol (30%–70%) and then embedded in paraffin. Hematoxylin and eosin were applied to stain tissue slices. Intestinal structures were observed using a microscope (Eclipse Ni-E, Nikon, Japan). The morphology parameters of the intestines were quantified with Image J software.

### 2.8. Calculations and Statistical Analysis

Parameters involved in growth performance, feed utilization, biometric parameters, and protein quality evaluation were calculated as follows:

(1) $$\begin{array}{c} \text{Initial body weight }\left(\text{IBW, g}\right)=\frac{\text{initial total body weight}}{\text{initial amount of fish}}, \end{array}$$

(2) $$\begin{array}{c} \text{Final body weight }\left(\text{FBW},g\right)=\frac{\text{final total body weight}}{\text{final amount of fish}}, \end{array}$$

(3) $$\begin{array}{c} \text{Weight gain ratio(WG, \%)}=100\times \frac{\left(\text{final body weight}-\text{initial body weight}\right)}{\text{initial body weight}}, \end{array}$$

(4) $$\begin{array}{c} \text{Specific growth ratio}\left(\text{SGR, \%/day}\right)=100\times \frac{\left(\text{Ln final body weight}-\text{Ln initial body weight}\right)}{\text{number of days}}, \end{array}$$

(5) $$\begin{array}{c} \text{Feed intake }\left(\text{FI, g/day/fish}\right)=\text{total feed intake}/\text{final amount of fish}/\text{number of days}, \end{array}$$

(6) $$\begin{array}{c} \text{Feed conversion ratio (FCR)}=\frac{\text{total feed intake}}{\text{total weight gain}}, \end{array}$$

(7) $$\begin{array}{c} \text{Survival rate (SR, \%)}=100\times \frac{\text{final amount of fish}}{\text{initial amount of fish}}, \end{array}$$

(8) $$\begin{array}{c} \text{Condition factor }\left(\text{CF},g/cm^{3}\right)=100\times \frac{\text{body weight}}{{\text{(body length)}}^{3}}, \end{array}$$

(9) $$\begin{array}{c} \text{Viscera-somatic index }\left(\text{VSI},\%\right)=100\times \frac{\text{visceral weight}}{\text{whole body weight}}, \end{array}$$

(10) $$\begin{array}{c} \text{Hepato-somatic index }\left(\text{HSI},\%\right)=100\times \frac{\text{hepatic weight}}{\text{whole body weight}}, \end{array}$$

(11) $$\begin{array}{c} \text{Proportion of essential amino acid (EAA)}=\frac{\text{essential amino acid content}}{\text{total amino acid content}}, \end{array}$$

(12) $$\begin{array}{c} \text{Ratio of amino acid (RAA)}=\text{EAA value of evaluated essential amino acid}/\text{corresponding EAA value in WHO}/\text{FAO essential amino acid requirement pattern}, \end{array}$$

(13) $$\begin{array}{c} \text{Ratio coefficient of amino acid (RC)}=\frac{\text{RAA}}{\text{mean of RAA}}, \end{array}$$

(14) $$\begin{array}{c} \text{Score of ratio coefficient of amino acid (SRC)}=100-\text{CV}\times 100;\text{CV was the coefficient of variation of RC}. \end{array}$$

The data were analyzed using one-way ANOVA (SPSS 26.0), and the results were presented as means ± SEM (standard error of the mean). Duncan's multiple test was applied to detect a difference between treatments at a significance level (*α* = 0.05).

## 3. Results

### 3.1. Growth Performance

**Figure 1:**
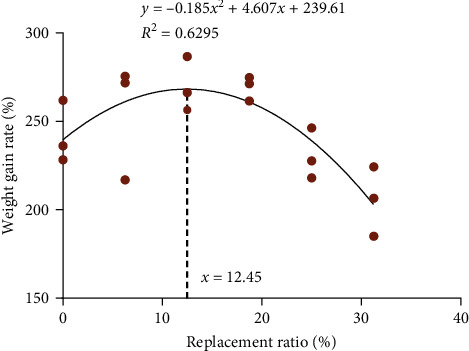
Regression analysis of replacement level and weight gain rate.

The growth performance of juvenile *T. ovatus* was summarized in Table 4. Fish given TD3 and TD4 diets performed better growth (FBW, WGR, and SGR) than those given TD5 and TD6 diets (*P* < 0.05), but comparable to those of fish fed TD1 and TD2 diets (*P* > 0.05). The regression analysis showed that the optimal WGR was obtained at a substitution level of 12.45% (Figure 1). The smallest FCR was observed in the TD4 group. There were no significant differences in the survival rate (SR) and CF among all experimental groups (*P* > 0.05). In contrast to the control group, a remarkable increase in VSI appeared in the TD2, TD4, TD5, and TD6 groups, and significantly higher HSI were observed in the TD3∼TD6 groups (*P* < 0.05).

### 3.2. Whole-Body and Muscle Compositions

As presented in Table 5, golden pompano juveniles supplied with TD5 and TD6 diets exhibited markedly lower whole-body moisture than fish given the control diet (*P* < 0.05). The whole-body crude protein was notably higher in fish supplied with the TD5 diet than those supplied with the TD1 diet (*P* < 0.05). There was no significant difference in the crude lipids of the whole body across all groups (*P* > 0.05). As for muscle, the replacement of fishmeal by ECGGR did not affect the proximate compositions among all groups (*P* > 0.05).

### 3.3. Muscle Amino Acid Profile and Nutritional Evaluation

The amino acid compositions of the muscles of golden pompano juveniles are shown in Table 6. As far as the essential amino acids (EAAs), the content of Ile in the TD3 group significantly increased compared to that in the TD1 group (*P* < 0.05), while the levels of Thr, Val, Met, Phe, Leu, and Lys were unaltered in all ECGGR groups compared to the control group (*P* > 0.05). What is more, the contents of total EAAs, total nonessential amino acids (NEAAs), and total flavor amino acids (FAAs) did not evidently differ across all groups (*P* > 0.05). However, the proportions of EAAs in total amino acids (*E*/*T*) were higher in the TD2, TD3, and TD6 diet treatments compared with the TD1 diet treatment (*P* < 0.05).

The nutritional values of the muscle protein of juvenile *T. ovatus* were evaluated according to the WHO/FAO EAA requirement pattern (Tables 7 and 8). The SRC values of all groups ranged from 78 to 80. Compared to the TD1 group, the SRC values of the TD2 and TD3 groups were significantly increased, whereas the other treatments exhibited comparable results (*P* > 0.05).

### 3.4. Hepatic Antioxidant Status

Hepatic antioxidant statuses are presented in Table 9. There were no significant changes in T-SOD, CAT, T-AOC, or MDA across all groups (*P* > 0.05). However, GSH-PX activity in fish given the TD4 diet was substantially lower than in those fed the TD1 diet (*P* < 0.05).

### 3.5. Hepatic Antioxidation-Related Genes

**Figure 2:**
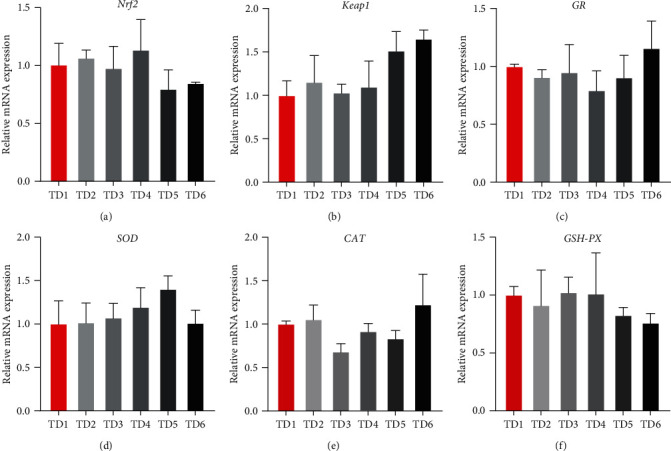
Expression levels of hepatic antioxidation-related genes. Nrf2, nuclear factor erythroid-2-related factor 2; Keap1, kelch-like ECH-associated protein 1; GR, glutathione reductase; SOD, superoxide dismutase; CAT, catalase; GSH-PX, glutathione peroxidase.

The expression levels of hepatic antioxidation-related genes are depicted in Figure 2. There were no significant differences in the relative expression levels of *Nrf2*, *Keap1*, *GR*, *SOD*, *CAT*, and *GSH-PX* across all groups (*P* > 0.05).

### 3.6. Intestinal Immune and Inflammatory Response-Related Genes

**Figure 3:**
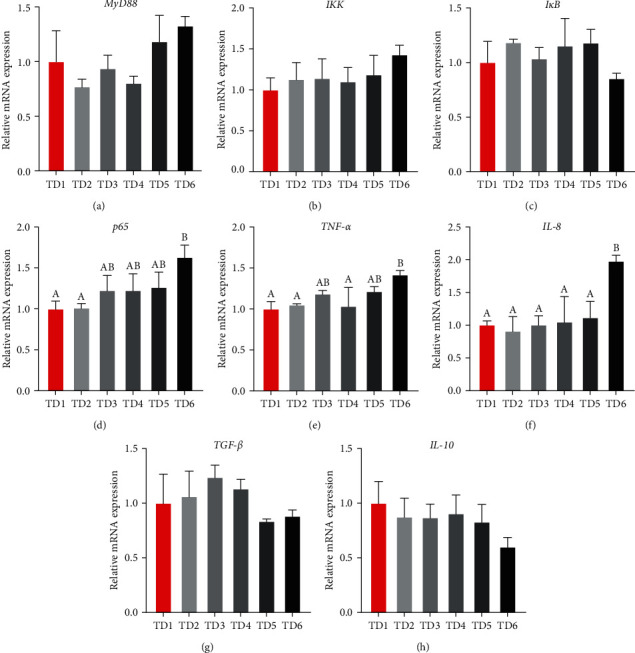
Expression levels of intestinal immune and inflammatory response-related genes. MyD88, myeloid differentiation factor 88; IKK, I*κ*B kinase; I*κ*B, NF-*κ*B inhibitor; p65, NF-*κ*B p65 protein; TNF-*α*, tumor necrosis factor-*α*; IL-8, interleukin-8; TGF-*β*, transforming growth factor-*β*; IL-10, interleukin-10.

As illustrated in Figure 3, relative expression levels of *MyD88*, *IKK*, *IκB*, *TGF-β*, and *IL-10* exhibited an insignificant difference among all dietary treatments (*P* > 0.05). Nevertheless, in comparison with the control group, the expressions of *p65*, *TNF-α*, and *IL-8* were upregulated in the TD6 group (*P* < 0.05).

### 3.7. Intestinal Morphology

**Figure 4:**
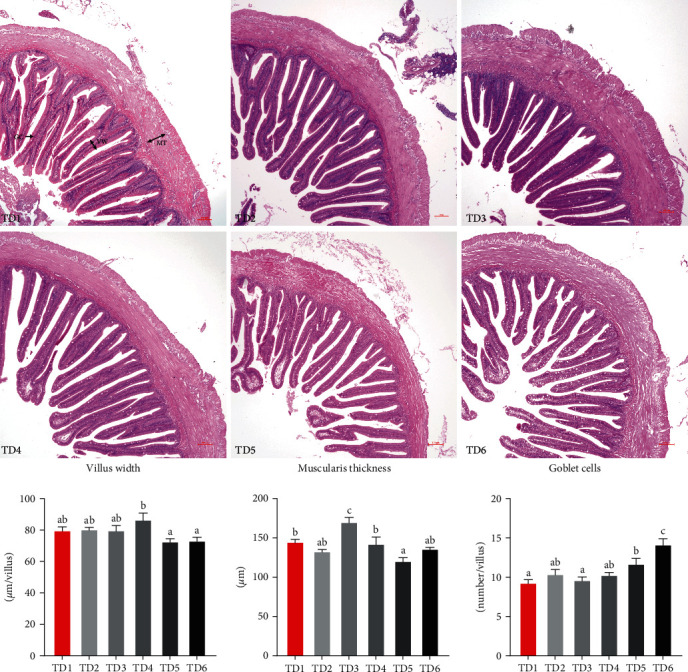
Microscopy of intestinal morphology and analysis of parameters. VW, villus width; MT, muscularis thickness; GC, goblet cells.

Intestinal morphology is shown in Figure 4. The intestinal villus widths in the TD2–TD6 treatments were similar to those of the control treatment (*P* > 0.05). In addition, a substantially increased intestinal muscularis thickness was observed in the TD3 group (*P* < 0.05), but the intestinal muscularis thickness notably decreased in the TD5 group (*P* < 0.05). More goblet cells were counted in the TD5 and TD6 groups compared to the control group (*P* < 0.05).

## 4. Discussions

The scarcity of fishmeal constrains the further development of the aquaculture industry, and hence, massive efforts have been spent to research alternative protein sources in the past decades. A suitable alternative protein source must be economical and not impair the growth of aquatic animals. The results of the present study indicated that the growth performance (FBW, WGR, and SGR) and SR of juvenile *T. ovatus* were not harmed when the ECGGR replacement level was no more than 25%. ECGGR is not only the residue ofa traditional Chinese medicine but also a kind of poultry byproduct. Previous studies have reported similar results on the substitution of PBM for fishmeal in other species. Riche et al. [29] reported that Florida pompano *Trachinotus carolinus* supplied with refined and blended poultry byproducts substituted for 66.7% fishmeal achieved comparable growth performance. Irm et al. [30] indicated that black sea bream had great tolerance for PBM supplementation when the replacement level was up to 30%. Chicken intestinal hydrolysate, as a kind of poultry byproduct, was reported as a promising candidate protein source used in common carp diets to replace 50% of fishmeal [18]. Previous studies also demonstrated that an excessive level of substitution of PBM for fishmeal gave rise to inhibition of growth performance [3, 31–35]. The optimal replacement level of poultry byproducts for fishmeal is determined by fish species, diet formulations, and the origins and processing methods of PBM. In the present study, 25% was the highest replacement level of ECGGR for fishmeal. Quadratic polynomial regression analysis suggested that fish could achieve the best WGR when ECGGR was substituted for 12.45% of fishmeal. However, with full results considered, the recommended replacement level was 18.75%.

Except for slight changes in whole-body moisture and crude protein, substitution of ECGGR for fishmeal had little influence on the whole-body crude lipid and proximate composition of muscles, which is consistent with other research on black seabream [30], gilthead seabream [36], red drum [37], Florida pompano [33], mirror crap [31], sobaity seabream [38], red porgy [39], and golden pompano [40]. The quality of aquatic products is inextricably linked to the amino acid composition of flesh. The current investigation indicated that substituting ECGGR for fishmeal did not adversely affect the contents of total amino acids, total EAAs, total NEAAs, total FAAs, or proportions of EAAs in total amino acids (*E*/*T*), although slight changes appeared in the contents of Gly, Cys, Ile, Tyr, and Pro. This outcome is accorded with previous findings on other species [7, 41, 42]. As a kind of nourishing food, the flesh of fish has a balanced amino acid composition for human health. FAO and WHO suggested an ideal EAA requirement pattern in 1973 [43]. The method *Ratio Coefficient of Amino Acid* was applied to evaluate the nutritional values of proteins [44, 45]. The value of the ratio coefficient of amino acid (RC) indicates whether the proportion of target amino acid in total amino acids conforms to the FAO/WHO pattern, while a value greater than 1 indicates an excess of the amino acid, and in contrast, a value less than 1 indicates deficiency. SRC is an index to measure the consistency of EAA composition in food with the FAO/WHO amino acid pattern, while 100 presents that the EAA composition of the food perfectly matches the ideal EAA requirement pattern. The present results demonstrated that substituting ECGGR for fishmeal did not impair the protein quality of the flesh of *T. ovatus*.

Since animal welfare is an important concern in animal production, a substitutive protein source must not compromise the health of fish. The expression levels of genes involved in the Nrf2-ARE pathway and relevant indices were measured to assess antioxidant status. Normally, Nrf2 is repressed in the cytoplasm by Keap1, which facilitates ubiquitination and eventual proteasomal degradation of Nrf2. Once oxidative stress is detected, the ubiquitin-proteasome system disrupts the coupling between Nrf2 and Keap1 [46, 47]. Nrf2 then translocates into the nucleus, associates with the sMaf protein, and binds to antioxidant-responsive elements (ARE), triggering the generation of phase II cytoprotective proteins such as superoxide dismutase [48]. The substitution of ECGGR for fishmeal had no impact on the relative expression levels of *Nrf2*, *Keap1*, *SOD*, *CAT*, *GSH-PX*, and *GR* in this work. T-AOC represents the overall level of enzymatic and non-enzymatic antioxidants. MDA is generated from lipid peroxidation and can cross-link with proteins and nucleic acids, contributing to cell and tissue damage. Except for slight changes in GSH-PX activity, the substitution of ECGGR for fishmeal did not affect the activities of T-SOD and CAT or the levels of MDA and T-AOC. Similarly, Wu et al. [18] indicated that the substitution of chicken intestinal hydrolysates at an appropriate level did not affect the activities of T-SOD, CAT, GSH-PX, or the content of MDA, as well as the expression levels of *CAT*, *Keap1*, and *Nrf2* in common carp. Zhang et al. [49] demonstrated that replacing fishmeal with yellow mealworm did not affect the antioxidative response of large yellow croakers. However, antioxidant status and antioxidant-related gene expression levels were altered in many other studies [22, 50–55]). In this study, the results indicated that substituting ECGGR for fishmeal did not cause oxidative stress in the livers of juvenile *T. ovatus*.

Organ health is a key factor in evaluating whether ECGGR is a suitable substitute for fishmeal. The TLR pathway and the NF-*κ*B pathway are classical pathways involved in immune and inflammatory responses. MyD88 is a critical component of the TLR pathway. It can interact with toll-like receptors and trigger the NF-*κ*B pathway. In the cytoplasm of most cells, NF-*κ*B/Rel usually binds to I*κ*B and is suppressed. Once receiving stimuli, the organism triggers signal transduction pathways and activates the I*κ*B kinase (IKK), leading to the phosphorylation of I*κ*Bs, which targets I*κ*Bs for ubiquitination and degradation. Following the degradation of I*κ*Bs, the NF-*κ*B/Rel complex translocates to the nucleus and binds to DNA to activate transcription [48, 56]. In the present study, the exceeded replacement level led to the upregulation of the expression levels of *p65* (*RelA*) as well as the pro-inflammatory cytokines *TNF-α* and *IL-8*. Meanwhile, the expression levels of other components of the NF-*κ*B pathway, *MyD88*, *IKK*, and *IκB*, as well as the anti-inflammatory cytokines *TGF-β* and *IL-10*, were maintained while the substitution levels varied. The overall result indicates that the excessive substitution of ECGGR for fishmeal led to an inflammatory response in the intestines. This is consistent with some of the findings of the previous research. Huang et al. [57] reported that excessive substitution of black soldier fly for fishmeal negatively affected the intestinal health of pearl gentian grouper. Li et al. [58] demonstrated that intestinal pathological changes were detected in Jian carp when defatted black soldier fly was substituted for more than 75% fishmeal, showing the upregulation of expression of *Hsp70* in the intestines. It has also been reported that upregulation of expressions of pro-inflammatory genes and downregulation of expressions of anti-inflammatory genes appeared in the livers of largemouth bass, presumably due to an excessive substitutive level (60%) of cottonseed protein concentrate for fishmeal [50]. The reasons for the inflammatory response vary with different substitutions, and some special antinutritional factors, for instance, chitin, raffinose, etc., may be responsible for that. The mechanism by which the substitution of ECGGR caused intestinal inflammation in this work remains to be further investigated.

Intestinal morphology observation is an effective means of evaluating the potential effects of diets on the intestines [59]. The width of the intestinal villus was positively associated with absorption capacity. Muscularis thickness was closely correlated to the ability of intestinal peristalsis. Goblet cells can synthesize and secrete Mucin 2 protein into intestinal mucus in response to stimuli [60]. Consistent with the above results of intestinal immune and inflammatory responses, the current result of intestinal morphology indicated an appropriate replacement level (less than 25%) of fishmeal with ECGGR did not impair the digestion and absorption capacity or lead to enteritis, but an excessive replacement level did. In line with our results, intestinal injuries caused by excessive replacement of fishmeal were found in largemouth bass [50], turbot [61], pearl gentian grouper [57], and common sole [54]. Therefore, it is crucial to regulate substitutive levels within appropriate limits to prevent any detrimental effects on the structure and function of the intestines.

## 5. Conclusions

In summary, ECGGR can be a substitute for fishmeal at an optimal level (18.75%) without adversely affecting growth performance, feed utilization, protein quality, antioxidant capacity, and intestinal health of juvenile golden pompano. ECGGR is a novel promising substitute for fishmeal in *T. ovatus* diet.

## Figures and Tables

**Table 1:** Proximate composition and amino acid profile of ECGGR.

Items	Content (g/kg)
Moisture	90.1 ± 0.7
Crude protein	839.3 ± 0.2
Crude lipid	15.9 ± 0.1
Aspartic acid	94.6 ± 1.1
Glutamic acid	113 ± 1.4
Threonine	37.4 ± 0.6
Serine	30.2 ± 0.5
Glycine	41.8 ± 0.5
Alanine	48.6 ± 0.4
Cysteine	9.6 ± 0.1
Valine	54.9 ± 0.8
Methionine	10.8 ± 0.1
Isoleucine	42.0 ± 0.7
Leucine	66.3 ± 0.9
Tyrosine	52.5 ± 0.4
Phenylalanine	41.6 ± 0.6
Histidine	16.3 ± 0.4
Lysine	25.4 ± 0.4
Arginine	76.8 ± 0.2
Proline	24.0 ± 0.3

**Table 2:** Composition and nutrition levels of the experimental diets (%, DM basis).

Ingredients	TD1 (0.00%)	TD2 (6.25%)	TD3 (12.5%)	TD4 (18.75%)	TD5 (25.00%)	TD6 (31.25%)
Fishmeal	32	30	28	26	24	22
ECGGR	0	2	4	6	8	10
Soybean meal	17	17.9	19	20	21	22
Wheat meal	22.23	22.23	22.23	22.23	22.23	22.23
Fish oil	5	5	5	5	5	5
Soy oil	5	5.1	5.2	5.3	5.4	5.5
Soy protein isolate	10	9.1	8	7	6	5
Soybean lecithin	2	2	2	2	2	2
Ca (H_2_PO_4_)_2_	1	1	1	1	1	1
Vitamin premix^a^	1	1	1	1	1	1
Mineral premix^b^	1	1	1	1	1	1
Choline chloride (50%)	0.5	0.5	0.5	0.5	0.5	0.5
Vitamin C	0.5	0.5	0.5	0.5	0.5	0.5
DL-Methionine	0.3	0.33	0.36	0.39	0.42	0.45
L-Lys-HCL (99%)	0.24	0.32	0.4	0.48	0.57	0.65
L-Threonine	0	0.03	0.07	0.1	0.14	0.17
Sodium alginate	1	1	1	1	1	1
Carboxymethyl cellulose	1.23	0.99	0.74	0.5	0.24	0
Sum	100	100	100	100	100	100
Nutrition levels^c^						
Moisture	8.98 ± 0.05	8.29 ± 0.13	10.51 ± 0.11	9.89 ± 0.08	10.25 ± 0.09	8.22 ± 0.20
Crude protein	44.39 ± 0.4	44.23 ± 0.24	44.09 ± 0.57	44.78 ± 0.69	44.75 ± 0.25	44.39 ± 0.37
Crude lipid	15.17 ± 0.24	15.32 ± 0.28	15.41 ± 0.26	15.09 ± 0.26	15.61 ± 0.30	15.26 ± 0.39

^a^Vitamin premix provides the following per kg of diet: thiamin, 25 mg; riboflavin, 45 mg; pyridoxine HCl, 20 mg; vitamin B12, 0.1 mg; vitamin K3,10 mg; inositol, 800 mg; pantothenic acid, 60 mg; niacin acid, 200 mg; folic acid, 20 mg; biotin, 1.20 mg; retinal acetate, 32 mg; cholecalciferol, 5 mg; tocopherols, 120 mg; ascorbic acid, 2,000 mg; choline chloride, 2,500 mg; ethoxyquin, 150 mg; wheat middling, 14.012 g. ^b^Mineral premix provides the following per kg of diet: NaF, 2 mg; KI, 0.8 mg; CoCl_2_ · 6H_2_O (1%), 50 mg; CuSO_4_ · 5H_2_O, 10 mg; FeSO_4_ · H_2_O, 80 mg; ZnSO_4_ · H_2_O, 50 mg; MnSO_4_ · H_2_O, 60 mg; MgSO_4_ · 7H_2_O, 1,200 mg; Ca (H_2_PO_4_)_2_·H_2_O, 3,000 mg; NaCl, 100 mg; zeolite,15.447 g. ^c^Measured values.

**Table 3:** Sequences of primers used in this study.

Gene	Primer sequence (5′−3′)	Reference
*Beta-actin*	F: TACGAGCTGCCTGACGGACA R: GGCTGTGATCTCCTTCTGCA	[24]
*Nrf2*	F: TTGCCTGGACACAACTGCTGTTAC R: TCTGTGACGGTGGCAGTGGAC	[24]
*Keap1*	F: CAGATAGACAGCGTGGTGAAGGC R: GACAGTGAGACAGGTTGAAGAACTCC	[24]
*HO-1*	F: AGAAGATTCAGACAGCAGCAGAACAG R: TCATACAGCGAGCACAGGAGGAG	[24]
*GR*	F: CCAGTGGCAATGAGGATGTGAGG R: ATTCAAGGTCACGCCAGGTTCAC	[24]
*SOD*	F: CCTCATCCCCCTGCTTGGTA R: CCAGGGAGGGATGAGAGGTG	[24]
*CAT*	F: GGATGGACAGCCTTCAAGTTCTCG R: TGGACCGTTACAACAGTGCAGATG	[24]
*GSH-PX*	F: GCTGAGAGGCTGGTGCAAGTG R: TTCAAGCGTTACAGCAGGAGGTTC	[24]
*MyD88*	F: AATACCTTGACAGCGATGCCTG R: GTGCAAGGCCTGGTGTAATCA	[25]
*IKK*	F: CCTGGAGAACTGCTGTGGAATGAG R: ATGGAGGTAGGTCAGAGCCGAAG	[26]
*IκB*	F: GCTGGTCCATTGCCTCCTGAAC R: GTGCCGTCTTCTCGTACAACTGG	[26]
*p65*	F: CGTGAGGTCAGCGAGCCAATG R: ATGTGCCGTCTATCTTGTGGAATGG	[26]
*TNF-α*	F: CGCAATCGTAAAGAGTCCCA R: AAGTCACAGTCGGCGAAATG	[27]
*IL-8*	F: TGCATCACCACGGTGAAAAA R: GCATCAGGGTCCAGACAAATC	[28]
*IL-10*	F: CTCCAGACAGAAGACTCCAGCA R: GGAATCCCTCCACAAAACGAC	[28]
*TGF-β*	F: GAGATACGGAAAAGAGTGGGG R: TGACAAAGCGGGAAGCAAG	[28]

**Table 4:** Growth performance, feed utilization, and biometric parameters of *T. ovatus*.

Items	TD1 (0%)	TD2 (6.25%)	TD3 (12.5%)	TD4 (18.75%)	TD5 (25%)	TD6 (31.25%)	*P*-value	
							Linear	Quadratic
IBW (g)	13.28 ± 0.10	13.36 ± 0.03	13.25 ± 0.08	13.36 ± 0.01	13.22 ± 0.05	13.28 ± 0.03	0.530	0.766
FBW (g)	45.43 ± 1.26bc	47.42 ± 2.61bc	48.99 ± 0.98c	49.34 ± 0.50c	43.71 ± 1.14ab	40.54 ± 1.45a	0.054	<0.001
WGR (%)	242.13 ± 10.15bc	254.80 ± 18.95bc	269.87 ± 8.85c	269.23 ± 3.95c	230.69 ± 8.29ab	205.29 ± 11.36a	0.059	0.001
SGR (%/day)	2.32 ± 0.06bc	2.38 ± 0.10bc	2.47 ± 0.04c	2.46 ± 0.02c	2.26 ± 0.05ab	2.10 ± 0.07a	0.053	0.001
SR (%)	81.11 ± 2.94	74.44 ± 6.76	74.44 ± 2.94	84.44 ± 1.11	84.44 ± 2.94	80.00 ± 5.09	0.342	0.622
FI (g/day/fish)	1.22 ± 0.01c	1.34 ± 0.00d	1.40 ± 0.01e	1.16 ± 0.01b	1.18 ± 0.04bc	1.10 ± 0.02a	0.008	0.001
FCR	1.43 ± 0.05b	1.50 ± 0.09b	1.52 ± 0.02b	1.25 ± 0.02a	1.44 ± 0.06b	1.43 ± 0.03b	0.524	0.707
CF (g/cm^3^)	3.45 ± 0.06	3.30 ± 0.08	3.45 ± 0.07	3.45 ± 0.11	3.48 ± 0.07	3.34 ± 0.05	0.951	0.823
VSI (%)	5.92 ± 0.16a	6.69 ± 0.22b	6.31 ± 0.08ab	6.74 ± 0.17b	6.46 ± 0.48b	6.60 ± 0.14b	0.038	0.028
HSI (%)	1.25 ± 0.04a	1.35 ± 0.06ab	1.60 ± 0.07c	1.67 ± 0.08c	1.49 ± 0.06bc	1.61 ± 0.09c	0.001	<0.001

Values in the same row with different letters are significantly different (*P*  < 0.05).

**Table 5:** Proximate compositions of the whole body and muscle of *T. ovatus*.

Items	TD1 (0%)	TD2 (6.25%)	TD3 (12.5%)	TD4 (18.75%)	TD5 (25%)	TD6 (31.25%)	*P*-value	
							Linear	Quadratic
Whole body								
Moisture (%)	68.97 ± 0.96b	67.38 ± 1.00ab	67.01 ± 0.53ab	67.76 ± 0.31ab	66.62 ± 0.31a	66.62 ± 0.60a	0.029	0.073
Crude protein (%)	16.57 ± 0.18a	17.02 ± 0.33ab	17.11 ± 0.19ab	17.10 ± 0.18ab	17.64 ± 0.21 b	17.33 ± 0.32ab	0.011	0.028
Crude lipid (%)	10.25 ± 0.59	11.28 ± 0.67	11.69 ± 0.45	10.75 ± 0.23	11.46 ± 0.34	11.58 ± 0.40	0.137	0.262
Muscle								
Moisture (%)	72.94 ± 0.14	73.17 ± 0.88	72.44 ± 0.45	71.85 ± 0.10	73.01 ± 0.34	73.50 ± 0.64	0.712	0.181
Crude protein (%)	18.83 ± 0.08	18.68 ± 0.61	19.47 ± 0.21	19.72 ± 0.36	19.45 ± 0.31	19.42 ± 0.22	0.066	0.098
Crude lipid (%)	6.25 ± 0.16	6.05 ± 0.34	6.57 ± 0.17	6.45 ± 0.24	6.10 ± 0.25	5.89 ± 0.50	0.479	0.336

Values in the same row with different letters are significantly different (*P*  < 0.05).

**Table 6:** Contents of amino acids in the muscle of *T. ovatus* (DM basis, mg/g).

Items	TD1 (0%)	TD2 (6.25%)	TD3 (12.5%)	TD4 (18.75%)	TD5 (25%)	TD6 (31.25%)	*P*-value	
							Linear	Quadratic
Asp^#^	64.47 ± 0.38	61.81 ± 0.46	64.53 ± 2.43	63.34 ± 2.69	63.51 ± 1.02	60.80 ± 1.63	0.306	0.477
Thr^*∗*^	27.09 ± 0.27	25.78 ± 0.19	26.79 ± 0.88	26.20 ± 0.88	26.29 ± 0.52	25.26 ± 0.93	0.153	0.347
Ser	25.82 ± 0.33	23.98 ± 0.46	24.89 ± 0.69	25.57 ± 0.91	25.48 ± 0.57	23.71 ± 0.92	0.407	0.629
Glu^#^	103.51 ± 1.30	96.34 ± 2.13	100.54 ± 4.12	100.29 ± 4.02	99.98 ± 3.08	92.93 ± 5.51	0.174	0.358
Gly	36.99 ± 0.52b	33.14 ± 0.59a	34.49 ± 1.62ab	35.68 ± 0.23ab	35.54 ± 0.75ab	34.65 ± 1.58ab	0.734	0.673
Ala	45.38 ± 0.29	42.51 ± 0.59	44.42 ± 1.90	44.79 ± 0.87	45.06 ± 0.94	42.13 ± 1.29	0.427	0.615
Cys	7.77 ± 0.14b	7.15 ± 0.30ab	7.17 ± 0.50ab	7.14 ± 0.52ab	7.07 ± 0.40ab	6.12 ± 0.12a	0.010	0.035
Val^*∗*^	28.89 ± 0.03	29.54 ± 0.32	31.92 ± 1.25	28.99 ± 1.70	30.09 ± 0.28	29.30 ± 0.65	0.929	0.415
Met^*∗*^	20.13 ± 0.12	19.43 ± 0.36	19.89 ± 1.02	19.86 ± 1.04	19.33 ± 0.85	18.02 ± 0.60	0.082	0.130
Ile^*∗*^	26.54 ± 0.26a	27.70 ± 0.29ab	30.26 ± 1.12b	27.17 ± 1.97ab	27.60 ± 0.44ab	27.57 ± 0.63ab	0.851	0.358
Leu^*∗*^	52.51 ± 0.51	52.16 ± 0.35	55.10 ± 1.96	52.80 ± 2.15	53.52 ± 0.80	51.40 ± 1.28	0.746	0.353
Tyr	21.69 ± 0.02b	20.16 ± 0.35ab	21.03 ± 0.95ab	20.73 ± 1.32ab	20.30 ± 0.37ab	19.15 ± 0.60a	0.050	0.138
Phe^*∗*^	25.88 ± 0.13	25.44 ± 0.25	26.61 ± 0.50	25.94 ± 1.22	26.19 ± 0.26	25.28 ± 0.67	0.778	0.541
His	17.87 ± 0.15	17.90 ± 0.30	18.66 ± 0.51	17.70 ± 0.48	18.13 ± 0.36	17.47 ± 0.85	0.577	0.490
Lys^*∗*^	60.59 ± 0.48	58.79 ± 0.52	62.28 ± 2.28	60.14 ± 2.75	60.41 ± 0.89	58.31 ± 1.57	0.531	0.525
Arg	41.07 ± 0.35	38.97 ± 0.60	41.12 ± 1.75	40.23 ± 1.32	40.21 ± 0.52	38.06 ± 1.07	0.194	0.299
Pro	15.57 ± 0.14b	13.38 ± 0.29a	13.72 ± 0.79a	14.57 ± 0.51ab	14.02 ± 0.50ab	13.05 ± 0.64a	0.075	0.195
TAAs	622.77 ± 5.11	596.20 ± 6.95	626.41 ± 23.72	615.15 ± 21.77	617.73 ± 11.66	589.22 ± 19.33	0.410	0.526
EAAs	241.63 ± 1.80	238.85 ± 1.87	252.84 ± 8.97	241.11 ± 11.11	243.43 ± 3.97	235.14 ± 6.05	0.587	0.410
NEAAs	381.15 ± 3.32	357.35 ± 5.21	373.57 ± 14.75	374.04 ± 11.28	374.30 ± 8.21	354.08 ± 13.44	0.350	0.588
FAAs	167.98 ± 1.67	158.16 ± 2.58	165.07 ± 6.39	163.63 ± 6.71	163.49 ± 4.03	153.73 ± 6.98	0.198	0.378
E (T) (%)	38.80 ± 0.03a	40.07 ± 0.19bc	40.37 ± 0.09c	39.17 ± 0.56ab	39.41 ± 0.32abc	39.93 ± 0.37bc	0.496	0.478

Values in the same row with different letters are significantly different (*P*  < 0.05).  ^*∗*^Means essential amino acids. ^#^Means flavor amino acids.

**Table 7:** Proportions of essential amino acids in the muscle of *T. ovatus* (DM basis, %).

Items	TD1 (0%)	TD2 (6.25%)	TD3 (12.5%)	TD4 (18.75%)	TD5 (25%)	TD6 (31.25%)	FAO/WHO pattern (1973)
Ile	4.26 ± 0.01	4.65 ± 0.05	4.83 ± 0.02	4.41 ± 0.22	4.47 ± 0.06	4.68 ± 0.05	4
Leu	8.43 ± 0.01	8.75 ± 0.05	8.80 ± 0.03	8.58 ± 0.06	8.67 ± 0.08	8.73 ± 0.09	7
Lys	9.73 ± 0.00	9.86 ± 0.08	9.94 ± 0.02	9.77 ± 0.11	9.78 ± 0.08	9.90 ± 0.06	5.5
Met + Cys	4.48 ± 0.04	4.46 ± 0.10	4.32 ± 0.08	4.39 ± 0.13	4.27 ± 0.11	4.10 ± 0.13	3.5
Phe + Tyr	7.64 ± 0.04	7.65 ± 0.02	7.61 ± 0.07	7.58 ± 0.13	7.53 ± 0.05	7.54 ± 0.05	6
Thr	4.35 ± 0.01	4.32 ± 0.02	4.28 ± 0.03	4.26 ± 0.03	4.26 ± 0.01	4.29 ± 0.02	4
Val	4.64 ± 0.03	4.95 ± 0.01	5.09 ± 0.02	4.71 ± 0.15	4.87 ± 0.06	4.98 ± 0.05	5

**Table 8:** Nutritional value of protein in muscle of *T. ovatus*.

Items	TD1 (0%)		TD2 (6.25%)		TD3 (12.5%)		TD4 (18.75%)		TD5 (25%)		TD6 (31.25%)	
	RAA	RC	RAA	RC	RAA	RC	RAA	RC	RAA	RC	RAA	RC
Ile	1.07 ± 0.00	0.87 ± 0.00	1.16 ± 0.01	0.92 ± 0.01	1.21 ± 0.00	0.95 ± 0.00	1.10 ± 0.06	0.89 ± 0.04	1.12 ± 0.02	0.90 ± 0.01	1.17 ± 0.01	0.94 ± 0.00
Leu	1.20 ± 0.00	0.98 ± 0.00	1.25 ± 0.01	0.99 ± 0.00	1.26 ± 0.00	0.99 ± 0.00	1.23 ± 0.01	0.99 ± 0.01	1.24 ± 0.01	1.00 ± 0.00	1.25 ± 0.01	1.00 ± 0.00
Lys	1.77 ± 0.00	1.44 ± 0.00	1.79 ± 0.01	1.42 ± 0.01	1.81 ± 0.00	1.43 ± 0.00	1.78 ± 0.02	1.44 ± 0.00	1.78 ± 0.02	1.44 ± 0.01	1.80 ± 0.01	1.45 ± 0.01
Met + Cys	1.28 ± 0.01	1.04 ± 0.01	1.27 ± 0.03	1.01 ± 0.02	1.23 ± 0.02	0.97 ± 0.02	1.25 ± 0.04	1.02 ± 0.03	1.22 ± 0.03	0.99 ± 0.03	1.17 ± 0.04	0.94 ± 0.02
Phe + Tyr	1.27 ± 0.01	1.04 ± 0.00	1.27 ± 0.00	1.01 ± 0.00	1.27 ± 0.01	1.00 ± 0.01	1.26 ± 0.02	1.02 ± 0.01	1.25 ± 0.01	1.02 ± 0.01	1.26 ± 0.01	1.01 ± 0.00
Thr	1.09 ± 0.00	0.88 ± 0.00	1.08 ± 0.01	0.86 ± 0.00	1.07 ± 0.01	0.84 ± 0.01	1.07 ± 0.01	0.86 ± 0.02	1.06 ± 0.00	0.86 ± 0.00	1.07 ± 0.00	0.86 ± 0.01
Val	0.93 ± 0.01	0.75 ± 0.00	0.99 ± 0.00	0.79 ± 0.00	1.02 ± 0.00	0.80 ± 0.00	0.94 ± 0.03	0.76 ± 0.02	0.97 ± 0.01	0.79 ± 0.01	1.00 ± 0.01	0.80 ± 0.01
SRC	78.11 ± 0.07a		79.50 ± 0.21b		79.62 ± 0.13b		78.15 ± 0.58a		78.84 ± 0.12ab		78.91 ± 0.19ab	

Values in the row SRC with different letters are significantly different (*P*  < 0.05).

**Table 9:** Hepatic antioxidant status of *T. ovatus*.

Items	TD1 (0%)	TD2 (6.25%)	TD3 (12.5%)	TD4 (18.75%)	TD5 (25%)	TD6 (31.25%)	*P*-value	
							Linear	Quadratic
T-SOD (U/mgprot)	823.68 ± 52.23	801.82 ± 9.74	843.08 ± 31.69	854.92 ± 38.30	781.74 ± 49.77	873.03 ± 44.97	0.556	0.811
CAT (U/mgprot)	11.20 ± 1.52	11.14 ± 0.96	10.56 ± 1.31	12.70 ± 1.40	10.37 ± 2.32	10.13 ± 1.65	0.656	0.770
GSH-PX (U/mgprot)	10.56 ± 0.63b	10.05 ± 0.20ab	10.67 ± 0.60b	8.03 ± 0.33a	9.86 ± 0.87ab	9.64 ± 0.96ab	0.227	0.329
T-AOC (mmol/gprot)	0.062 ± 0.003	0.062 ± 0.002	0.065 ± 0.001	0.063 ± 0.002	0.069 ± 0.001	0.064 ± 0.010	0.444	0.708
MDA (nmol/mgprot)	0.09 ± 0.01	0.17 ± 0.02	0.13 ± 0.04	0.10 ± 0.03	0.13 ± 0.03	0.18 ± 0.06	0.313	0.569

Values in the same row with different letters are significantly different (*P*  < 0.05).

## Data Availability

The data that support the findings of this study are available from the corresponding author upon reasonable request.

## References

[1] FAO (2022). *The State of World Fisheries and Aquaculture 2022*.

[2] FAO (2020). *The State of World Fisheries and Aquaculture 2020*.

[3] Karapanagiotidis I. T., Psofakis P., Mente E., Malandrakis E., Golomazou E. (2019). Effect of fishmeal replacement by poultry by-product meal on growth performance, proximate composition, digestive enzyme activity, haematological parameters and gene expression of gilthead seabream (*Sparus aurata*). *Aquaculture Nutrition*.

[4] Moutinho S., Peres H., Serra C. (2017). Meat and bone meal as partial replacement of fishmeal in diets for gilthead sea bream (*Sparus aurata*) juveniles: Diets digestibility, digestive function, and microbiota modulation. *Aquaculture*.

[5] Psofakis P., Karapanagiotidis I. T., Malandrakis E. E., Golomazou E., Exadactylos A., Mente E. (2020). Effect of fishmeal replacement by hydrolyzed feather meal on growth performance, proximate composition, digestive enzyme activity, haematological parameters and growth-related gene expression of gilthead seabream (*Sparus aurata*). *Aquaculture*.

[6] Wu F., Tian J., Yu L., Wen H., Jiang M., Lu X. (2021). Effects of dietary rapeseed meal levels on growth performance, biochemical indices and flesh quality of juvenile genetically improved farmed tilapia. *Aquaculture Reports*.

[7] Xu J., Sheng Z., Chen N., Xie R., Zhang H., Li S. (2022). Effect of dietary fish meal replacement with spray dried chicken plasma on growth, feed utilization and antioxidant capacity of largemouth bass (*Micropterus salmoides*). *Aquaculture Reports*.

[8] Yang P., Li X., Song B., He M., Wu C., Leng X. (2021). The potential of Clostridium autoethanogenum, a new single cell protein, in substituting fish meal in the diet of largemouth bass (*Micropterus salmoides*): growth, feed utilization and intestinal histology. *Aquaculture and Fisheries*.

[9] Ye H., Xu M., Liu Q. (2019). Effects of replacing fish meal with soybean meal on growth performance, feed utilization and physiological status of juvenile obscure puffer, *Takifugu obscurus*. *Comparative Biochemistry and Physiology Part C: Toxicology and Pharmacology*.

[10] Zhang Q., Liang H., Longshaw M. (2022). Effects of replacing fishmeal with methanotroph (*Methylococcus capsulatus*, Bath) bacteria meal (FeedKind®) on growth and intestinal health status of juvenile largemouth bass (*Micropterus salmoides*). *Fish & Shellfish Immunology*.

[11] Zhao W., Liu Z.-L., Niu J. (2021). Growth performance, intestinal histomorphology, body composition, hematological and antioxidant parameters of *Oncorhynchus mykiss* were not detrimentally affected by replacement of fish meal with concentrated dephenolization cottonseed protein. *Aquaculture Reports*.

[12] Chinese Pharmacopoeia Committee (2020). *The Pharmacopoeia of People’s Republic of China (IV)*.

[13] Li S., Zheng M., Zhang Z., Peng H., Dai W., Liu J. (2021). Galli gigeriae endothelium corneum: its intestinal barrier protective activity in vitro and chemical composition. *Chinese Medicine*.

[14] Wang N., Zhang D., Zhang Y.-T. (2019). Endothelium corneum gigeriae galli extract inhibits calcium oxalate formation and exerts anti-urolithic effects. *Journal of Ethnopharmacology*.

[15] Xiong Q., Jing Y., Li X. (2015). Characterization and bioactivities of a novel purified polysaccharide from Endothelium corneum gigeriae galli. *International Journal of Biological Macromolecules*.

[16] Xiong Q., Li X., Zhou R. (2014). Extraction, characterization and antioxidant activities of polysaccharides from E. corneum gigeriae galli. *Carbohydrate Polymers*.

[17] Xiangbing Z., Hongbiao D., Zhengkun W. (2021). Effects of polysaccharide from Endothelium corneum gigeriae galli on growth, digestive, intestinal antioxidant capacity and serum biochemical indices of *Lates calcarifer*. *South China Fisheries Science*.

[18] Wu D., Zhang Y., Li J., Fan Z., Xu Q., Wang L. (2022). Assessment of chicken intestinal hydrolysates as a new protein source to replace fishmeal on the growth performance, antioxidant capacity and intestinal health of common carp (*Cyprinus carpio*). *Fish & Shellfish Immunology*.

[19] Yang X., Dong X., Yang Q. (2021). Addition of enzyme-digested hydrolysed porcine mucosa to low-fishmeal feed improves growth, intestinal microbiota, and intestinal peptide and amino acid transporter expressions in hybrid groupers (*Epinephelus fuscoguttatus* ♀ × *E. lanceolatus* ♂). *Aquaculture Nutrition*.

[20] Zhou C., Ge X., Niu J., Lin H., Huang Z., Tan X. (2015). Effect of dietary carbohydrate levels on growth performance, body composition, intestinal and hepatic enzyme activities, and growth hormone gene expression of juvenile golden pompano, *Trachinotus ovatus*. *Aquaculture*.

[21] Fishery Administration of the Ministry of Agriculture and Rural Affairs, China Society of Fisheries, N.F.T.E.C. (2023). *China Fisheries Statistical Yearbook*.

[22] Ma Y., Li M., Xie D. (2020). Fishmeal can be replaced with a high proportion of terrestrial protein in the diet of the carnivorous marine teleost (*Trachinotus ovatus*). *Aquaculture*.

[23] AOAC (1995). *Official Methods of Analysis Of The Association Of Analytical Chemists*.

[24] Xie J., Niu J. (2022). Evaluation of four macro-algae on growth performance, anti-oxidant capacity and non-specific immunity in golden pompano (*Trachinotus ovatus*). *Aquaculture*.

[25] Xie J., Fang H., Liao S. (2019). Study on *Schizochytrium sp*. improving the growth performance and non-specific immunity of golden pompano (*Trachinotus ovatus*) while not affecting the antioxidant capacity. *Fish & Shellfish Immunology*.

[26] Xie J., Fang H., He X. (2020). Study on mechanism of synthetic astaxanthin and *Haematococcus pluvialis* improving the growth performance and antioxidant capacity under acute hypoxia stress of golden pompano (*Trachinotus ovatus*) and enhancing anti-inflammatory by activating Nrf2-ARE pathway to antagonize the NF-*κ*B pathway. *Aquaculture*.

[27] Zhou C., Lin H., Huang Z., Wang J., Wang Y., Yu W. (2020). Effects of dietary leucine levels on intestinal antioxidant status and immune response for juvenile golden pompano (*Trachinotus ovatus*) involved in Nrf2 and NF-*κ*B signaling pathway. *Fish & Shellfish Immunology*.

[28] Tan X., Sun Z., Huang Z. (2017). Effects of dietary hawthorn extract on growth performance, immune responses, growth-and immune-related genes expression of juvenile golden pompano (*Trachinotus ovatus*) and its susceptibility to *Vibrio harveyi* infection. *Fish & Shellfish immunology*.

[29] Riche M. (2015). Nitrogen utilization from diets with refined and blended poultry by-products as partial fish meal replacements in diets for low-salinity cultured Florida pompano, *Trachinotus carolinus*. *Aquaculture*.

[30] Irm M., Taj S., Jin M., Luo J., Andriamialinirina H. J. T., Zhou Q. (2020). Effects of replacement of fish meal by poultry by-product meal on growth performance and gene expression involved in protein metabolism for juvenile black sea bream (*Acanthoparus schlegelii*). *Aquaculture*.

[31] Emre Y., Sevgili H., Diler I. (2004). Replacing fish meal with poultry by-product meal in practical diets for mirror carp (*Cyprinus carpio*) fingerlings. *Turkish Journal of Fisheries and Aquatic Sciences*.

[32] Pham H. D., Siddik M. A. B., Phan U. V., Le H. M., Rahman M. A. (2021). Enzymatic tuna hydrolysate supplementation modulates growth, nutrient utilisation and physiological response of pompano (*Trachinotus blochii*) fed high poultry-by product meal diets. *Aquaculture Reports*.

[33] Rossi W., Davis D. A. (2012). Replacement of fishmeal with poultry by-product meal in the diet of Florida pompano *Trachinotus carolinus* L. *Aquaculture*.

[34] Shapawi R., Ng W.-K., Mustafa S. (2007). Replacement of fish meal with poultry by-product meal in diets formulated for the humpback grouper, *Cromileptes altivelis*. *Aquaculture*.

[35] Wang Z., Qian X., Xie S., Yun B. (2020). Changes of growth performance and plasma biochemical parameters of hybrid grouper (*Epinephelus lanceolatus* ♂ × *Epinephelus fuscoguttatus* ♀) in response to substitution of dietary fishmeal with poultry by-product meal. *Aquaculture Reports*.

[36] Fontinha F., Magalhães R., Moutinho S. (2021). Effect of dietary poultry meal and oil on growth, digestive capacity, and gut microbiota of gilthead seabream (*Sparus aurata*) juveniles. *Aquaculture*.

[37] Suehs B. A., Alfrey K., Barrows F., Gatlin D. M. (2022). Evaluation of growth performance, condition indices and body composition of juvenile red drum (*Sciaenops ocellatus*) fed fishmeal- and fish-oil-free diets. *Aquaculture*.

[38] Hekmatpour F., Kochanian P., Marammazi J. G., Zakeri M., Mousavi S.-M. (2019). Changes in serum biochemical parameters and digestive enzyme activity of juvenile sobaity sea bream (*Sparidentex hasta*) in response to partial replacement of dietary fish meal with poultry by-product meal. *Fish Physiology and Biochemistry*.

[39] Hill J. C., Alam M. S., Watanabe W. O., Carroll P. M., Seaton P. J., Bourdelais A. J. (2019). Replacement of menhaden fish meal by poultry by-product meal in the diet of juvenile red porgy. *North American Journal of Aquaculture*.

[40] Ma X., Wang F., Han H., Wang Y., Lin Y. (2014). Replacement of dietary fish meal with poultry by-product meal and soybean meal for golden pompano, *Trachinotus ovatus*, reared in net pens. *Journal of the World Aquaculture Society*.

[41] Ma Y., Xu C., Li M. (2020). Diet with a high proportion replacement of fishmeal by terrestrial compound protein displayed better farming income and environmental benefits in the carnivorous marine teleost (*Trachinotus ovatus*). *Aquaculture Reports*.

[42] Sabbagh M., Schiavone R., Brizzi G., Sicuro B., Zilli L., Vilella S. (2019). Poultry by-product meal as an alternative to fish meal in the juvenile gilthead seabream (*Sparus aurata*) diet. *Aquaculture*.

[43] FAO/WHO Expert Committee (1973). Energy and protein requirements.

[44] Liu G., Wang H., Zhou B., Guo X., Hu X. (2010). Compositional analysis and nutritional studies of *Tricholoma matsutake* collected from Southwest China. *Journal of Medicinal Plants Research*.

[45] Zhu S., Wu K. (1956). Nutritional evaluation of protein——ratio coefficient of amino acid.

[46] Xie J., Chen X., Niu J. (2021). Cloning, functional characterization and expression analysis of nuclear factor erythroid-2-related factor-2 of golden pompano (*Trachinotus ovatus*) and its response to air-exposure. *Aquaculture Reports*.

[47] Xie J., Chen X., He X., Niu J. (2020). Cloning and expression analysis of Keap1 of golden pompano (*Trachinotus ovatus*) and response to oxidized fish oil and LPS administration. *Aquaculture Reports*.

[48] Wardyn J. D., Ponsford A. H., Sanderson C. M. (2015). Dissecting molecular cross-talk between Nrf2 and NF-*κ*B response pathways. *Biochemical Society Transactions*.

[49] Zhang Z., Yuan J., Tian S. (2022). Effects of dietary vitamin E supplementation on growth, feed utilization and flesh quality of large yellow croaker *Larimichthys crocea* fed with different levels of dietary yellow mealworm *Tenebrio molitor* meal. *Aquaculture*.

[50] He G., Zhang T., Zhou X. (2022). Effects of cottonseed protein concentrate on growth performance, hepatic function and intestinal health in juvenile largemouth bass, *Micropterus salmoides*. *Aquaculture Reports*.

[51] Hidalgo M. C., Morales A. E., Pula H. J. (2022). Oxidative metabolism of gut and innate immune status in skin and blood of tench (*Tinca tinca*) fed with different insect meals (*Hermetia illucens* and *Tenebrio molitor*). *Aquaculture*.

[52] Liu T., Han T., Wang J. (2021). Effects of replacing fish meal with soybean meal on growth performance, feed utilization and physiological status of juvenile redlip mullet *Liza haematocheila*. *Aquaculture Reports*.

[53] Liu X., Han B., Xu J. (2020). Replacement of fishmeal with soybean meal affects the growth performance, digestive enzymes, intestinal microbiota and immunity of *Carassius auratus gibelio*♀ × *Cyprinus carpio*♂. *Aquaculture Reports*.

[54] Saleh N. E., Mourad M. M., El-Banna S. G., Abdel-Tawwab M. (2021). Soybean protein concentrate as a fishmeal replacer in weaning diets for common sole (*Solea solea*) post-larvae: Effects on the growth, biochemical and oxidative stress biomarkers, and histopathological investigations. *Aquaculture*.

[55] Zhang X., Sun Z., Cai J. (2020). Effects of dietary fish meal replacement by fermented moringa (*Moringa oleifera* Lam.) leaves on growth performance, nonspecific immunity and disease resistance against *Aeromonas hydrophila* in juvenile gibel carp (*Carassius auratus* gibelio var. CAS III). *Fish & Shellfish Immunology*.

[56] Xie J., Liao S., Wang R. (2020). Molecular cloning, functional characterization and expression analysis of p65 subunit of golden pompano (*Trachinotus ovatus*) and response to high fat diet and LPS administration. *Aquaculture*.

[57] Huang B., Zhang S., Dong X. (2022). Effects of fishmeal replacement by black soldier fly on growth performance, digestive enzyme activity, intestine morphology, intestinal flora and immune response of pearl gentian grouper (*Epinephelus fuscoguttatus* ♀ × *Epinephelus lanceolatus* ♂). *Fish & Shellfish Immunology*.

[58] Li S., Ji H., Zhang B., Zhou J., Yu H. (2017). Defatted black soldier fly (*Hermetia illucens*) larvae meal in diets for juvenile Jian carp (*Cyprinus carpio* var. Jian): growth performance, antioxidant enzyme activities, digestive enzyme activities, intestine and hepatopancreas histological structure. *Aquaculture*.

[59] Hartviksen M., Vecino J. L. G., Ringø E. (2014). Alternative dietary protein sources for Atlantic salmon (*Salmo salar* L.) effect on intestinal microbiota, intestinal and liver histology and growth. *Aquaculture Nutrition*.

[60] López-Cauce B., Urquía A., Menchén L. (2022). *Lentinula edodes* extract increases goblet cell number and Muc2 expression in an intestinal inflammatory model of *Trichinella spiralis* infection. *Biomedicine & Pharmacotherapy*.

[61] Zhang X., Zhou H., Liu C., Mai K., He G., Wang X. (2022). Fishmeal substitution with low-gossypol cottonseed meal in the diet for juvenile turbot (*Scophthalmus maximus* L.): effects on growth, nutrients utilization and haematological responses. *Aquaculture Reports*.

